# Establishment of a facial nerve trunk crush injury model and evaluation of facial nerve self‐healing in rats

**DOI:** 10.1002/brb3.3156

**Published:** 2023-08-07

**Authors:** Jing Fei, Xirui Guan, Lin Gao, Ping Ni, Hongdi Zheng, Kunling Duan, Na Liao, Leiji Li

**Affiliations:** ^1^ Department of Otorhinolaryngology Head and Neck Surgery The Affiliated Hospital of Southwest Medical University Lu Zhou China; ^2^ Department of Health Management Center The Affiliated Hospital of Southwest Medical University Lu Zhou China; ^3^ Department of Otolaryngology The First People's Hospital of Yibin Yibin China

**Keywords:** disease models, facial nerve, facial paralysis, nerve regeneration, peripheral nerve injuries

## Abstract

**Introduction/aims:**

To facilitate further investigation into the mechanisms of facial nerve regeneration, a simple and reliable model of facial nerve crush injury is essential. Nevertheless, the establishment of such models lacks standardization and repeatability, while the healing capacity of the nerve is often overlooked, potentially affecting future studies.

**Methods:**

We made facial nerve trunk crush injury models with different pressing times and detected the changes from the distal nerves to the motoneurons via behavior analysis, electrophysiological test, and histomorphometry analysis.

**Results:**

It revealed a particular capacity for self‐healing following facial nerve crush damage because there was almost no facial motoneuron apoptosis in the MC group during the observation period, and rats in MC group had total facial paralysis in behavioral tests following surgery and varying degrees of recovery 28 days postoperatively with no treatments. As the pressing time increased, the latency, wave amplitude, nerve fiber damage degree, nerve axon ratio, myelin thickness, electroneurograph (ENoG) value, ultrastructural damage, abnormal morphological changes, and the buccal muscle atrophy of each MC group gradually increased or got worse during the observation period. However, after 28 postoperative days, only the ENoG values of the M10min and M12min groups were beyond 90%, indicating no self‐healing.

**Discussion:**

It suggests that a stable model of peripheral facial palsy may be created by applying a 12.5 cm mosquito clamped to the facial nerve trunk for at least 10 min, which laid the foundation for the subsequent research to objectively evaluate facial nerve regeneration.

## Graphical abstract

For subsequent researches on the mechanism of facial nerve regeneration, a straightforward and reliable model of facial nerve crush injury will serve as the foundation.In this work, the results suggested that a stable model of peripheral facial palsy may be created by applying a 12.5cm mosquito clamped to the facial nerve trunk for at least 10 minutes, which layed the foundation for the the subsequent researches to objectively evaluate facial nerve regeneration.

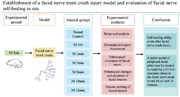

## 1 INTRODUCTION

As it affects 20–30 per 100,000 persons each year, the prevalence of facial nerve dysfunction can be rather high (Ali et al., 2020). In China, the incidence of facial palsy is 49.77 per 100,000 people 1 year (Neurophysiological Monitoring Group, 2022). In those who are affected, the loss of facial nerve function results in emotional and practical issues, including embarrassment, functional limitations, low self‐esteem, and a poor quality of life (Toraman et al., 2021). Although many studies have investigated the mechanisms of facial nerve damage and restoration, more research are necessary to achieve better outcomes. Developing a standardized facial nerve injury model would be of significant value to the otolaryngologists or plastic surgeons interested in facial nerve regeneration.

Currently, there is no standardized approach for creating facial palsy models, which can be produced using various methods, including compression (Cai et al., 2021), transection (Fujii et al., 2020), cold stimulation (Joko et al., 2020), and inoculation virus method (Mu et al., 2022). Peripheral nerve injury in maxillofacial surgery most frequently manifested as compression and contusion (Mourad et al., 2022). Hence, we believe that, unlike transection injury, the crush injury model produces consistent damage that is reproducible and clinically relevant. However, there is currently no standardized protocol regarding the age of animals used for modeling or the duration and extent of facial nerve entrapment.

On the other hand, most experimental researches on facial nerve injury rely solely on the compressed section of the facial nerve that was obtained during the modeling process or on the animal's symptoms to evaluate the effectiveness of the model (Zhang et al., 2012). If the self‐healing of the facial nerve compression model is not taken into account, the therapeutic efficacy of the model may be assessed solely based on the presence or absence of symptoms, which can affect subsequent stages of the study. Therefore, it is crucial to determine whether the facial palsy model undergoes self‐healing during the experiment.

A comprehensive analysis about the self‐healing of facial nerves after the compression injury from these aspects, including animal behavior, neuron electrophysiology, neurohistopathology, atrophy of target muscles, and apoptosis of central motoneurons, has not been reported at present. According to the previous literature, in our experiment, we set up six groups of different compression durations of the facial nerve trunk for 10 s, 1 min, 5 min, 8 min, 10 min, and 12 min. Through this approach, we were able to establish a reproducible and reliable compression model for further facial palsy research. This model will serve as a basis for studying the mechanisms underlying facial palsy recovery.

## 2 METHODS

### 2.1 Animals and groups

A total of 104 Sprague Dawley (SD) rats, both male and female, aged 4–5 months, weighing 250–300 g/each were provided by the Experimental Center of Southwest Medical University (Laboratory Animal Certificate No. SYXK(Chuan) 2018‐065). Feeding settings include general‐grade animal housing with humidity levels of 55%–55%, a temperature range of 22−24°C, 12‐h cycles of light and dark, and frequent ventilation, with separate cages housing a maximum of eight animals each. The entire experimental procedure was overseen by a single individual, and special animal feed was used. The rats were allowed to eat and drink whatever they wanted, except the 8‐h preoperative fast. The Ethics Association approved the operation of experimental animals at Southwest Medical University (No. SWMU20220093).

The 104 healthy adult SD rats were at random assigned to a normal control (NC, *n* = 8) group and six model groups: 10 seconds pressed group (M10s, *n* = 16), 1 min pressed group (M1min, *n* = 16), 5 min pressed group (M5min, *n* = 16), 8 min pressed group (M8min, *n* = 16), 10 min pressed group (M10min, *n* = 16), and 12 min pressed group (M12min, *n* = 16).

### 2.2 Surgical procedure

**FIGURE 1:**
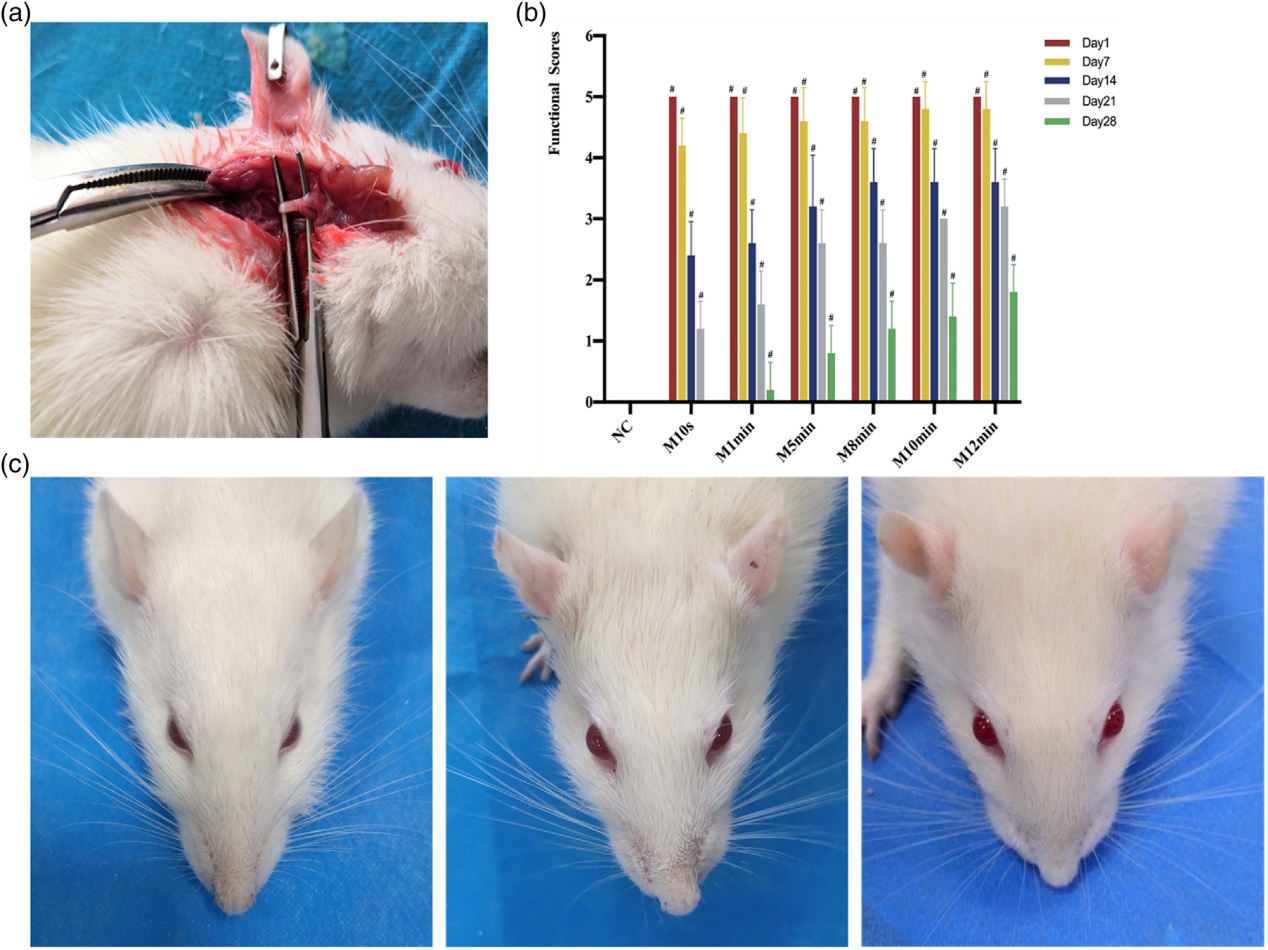
(a) The right facial nerve trunk was exposed in surgery. (b) Functional scores at different time points in each group. *Note*: The animal behavior in the normal control (NC) group received a score of 0 at all time points, while each MC group showed a gradual decrease in scores at each postoperative time point. As the pressing time was prolonged, the scores of each MC group gradually increased and showed significant differences between groups at each time point. There were no significant differences between the MC group at any time point. #*p* < 0.05 versus NC. (c) The performance of peripheral facial palsy in each group. *Note*: The placement of the apex nasi in NC group rats was in the center, and the beards on both sides are symmetrical and move normally. After surgery, rats in each MC group showed peripheral facial palsy with crooked mouth, skewed apex nasi, and weakened beard movement. At 28 days after surgery, the symptoms of facial palsy in each MC group nearly disappeared.

The surgical procedures were carried out in an aseptic environment. Ketamine (40 mg/kg) and xylazine (5 mg/kg) were used to anesthetize the rats intraperitoneally. After that, the facial nerve, which emerges from the stylomastoid foramen, was tightly clamped 2‐mm‐long section with a 12.5‐cm mosquito forcep held perpendicular to the nerve's longitudinal axis (Figure 1a). The trunk was continuously pressed for 10 s, 1 min, 5 min, 8 min, 10 min, and 12 min, respectively. After multilayer suture and disinfection, the incision was closed. Only the right facial nerve trunk was exposed in the NC group during surgery, and the incision was stitched together in layers.

### 2.3 Behavioral analysis

The behavior assessment was performed in each group on 1, 7, 14, 21, and 28 days postoperatively. Twice blowing air over the eyelids caused the blink reflex to occur with a 5‐mL syringe connected to an 18‐gauge needle, placed around 3 cm from the eyes. On a scale of 0–2, the blink reflex intensity was evaluated (0, no difference between the two sides; 1, a delay in the blink reflex in comparison to the unaffected side; 2, a complete disappearance of the blink reflex). After 30 s of observation, the vibrissae's movement was rated on a scale from 0 to 2 (0 signifies no change between the two sides; 1, a weaker vibrissae movement than on the healthy side; 2, a disappearance of vibrissae movement). A score between 0 and 1 was assigned for the positioning of the apex nasi (0 meant it was in the center; 1 told it was toward the unaffected side) (Cai et al., 2021). The final scores were calculated as the sum of all scores. The NC group had a total score of 0, showing no signs of facial nerve paralysis. Symptoms of facial paralysis were taken into account when the scores on aggregate were higher than 3 (Figure 1c).

### 2.4 Electrophysiological assessment

At 10 min and 28 days postoperatively, animals in each group were anesthetized for the electrophysiological tests. At the intersection of the facial nerve's buccal branch and the canthus vertical line, two stimulating electrodes were placed, and the collecting electrodes were positioned subcutaneously on the right side of the orbicularis oris muscle in order to measure the compound muscle action potentials (CMAPs) of the injured facial nerves. A reference electrode was placed subcutaneously on the tail. The parameters were adjusted as follows: a square wave with a 0.2 ms wavelength and a 2.5 V stimulation intensity. Then the peak amplitude and latent period in each rat were recorded. Normal CMAPs were obtained from the NC group.

The latency stage before facial nerve trunk injury was recorded as La. The latency stage after surgery was recorded as Lb, and 28 days postoperatively it was recorded as Lc. The peak value of M‐wave was represented as A. Before surgery, it was recorded as Aa, and it was recorded as Ab after injury. The peak value of the M‐wave was recorded as Ac 28 days postoperatively. The ENoG value after surgery equals (1 − Ab/Aa) × 100%, and the ENoG value 28 days postoperatively equals (1 − Ac/Aa) × 100% .

### 2.5 Tissue sample preparation

Rats (*n* = 4) were randomly selected from each group immediately following the surgery and 28 days postoperatively. Animals were transcardially perfused with 4% paraformaldehyde to be pre‐fixed under deep anesthesia. The facial nerve and buccal muscle tissues were collected, drained, made translucent, waxed, and embedded into paraffin blocks after being treated in 4% paraformaldehyde. Then the rats were placed on a brain stereotaxic instrument to determine the location of the facial motoneurons. The neck was broken, the temporal bone was opened, the brainstem was removed, and the brainstem tissues containing the facial motoneurons (up to below the inferior colliculus and down to the pontine sulcus) were cut off with a blade. Frozen sections were made, followed by continuous coronal sectioning and observation under a light microscope until the facial motoneurons appeared. The remaining rats (*n* = 4) from each group were transcardially perfused with 2.5% glutaraldehyde under deep anesthesia immediately following surgery and 28 days postoperatively for transmission electron microscopy (TEM). The facial nerves that had been removed were treated in 2.5% glutaraldehyde.

### 2.6 Pathological evaluation of facial nerve

Routine procedures with Hematoxylin‐Eosin (HE) staining included dewaxing and hydrating paraffin sections of the facial nerve, staining with hematoxylin for 10 min, rinsing in tap water for 5 min, fractionating in hydrochloric acid alcohol fractionation solution for 30 s, soaking in tap water for 3 min, returning to blue in warm water at 50°C, staining with 85% alcohol for 5 min, staining with eosin for 5 min, soaking in tap water for 5 min, dehydrating, transparenting, and sealing. Then we observed the samples under the light microscope.

For TEM, the facial nerve specimens were implanted to create ultrathin sections about 50 nm thick after being refixed in 1% osmium tetroxide, dried in acetone, and permeabilized by dehydrating agent epoxy resin in succession. Then uranium acetate staining was performed for 10 min, and lead citrate staining was carried out for 2 min at room temperature. Samples were photographed with a digital camera attached to the microscope. To evaluate the regeneration of the injured nerve, the myelin sheath thickness (maximum diameter of myelin sheath + minimum diameter)/2), the number of axons, and the ratio of normal axons (number of normal axons/total axons) were calculated. According to damage, scores for axonal ultrastructure were 0 (no damage), 1+ (low damage), 2+ (moderate damage), or 3+ (severe damage) (Ozbay et al., 2017).

### 2.7 Nissl staining of facial motoneuron

Frozen sections were defrosted, baked for 30 min at 37°C, submerged in 1% toluidine blue solution heated to 50°C, and stained for 20 min at 56°C. Then they were rinsed with distilled water, immersed in 70% alcohol for 1 min, differentiated in 95% alcohol, transparented, sealed, and inspected under the light microscopy.

### 2.8 Tunel assay of facial motoneuron

Brainstem tissues containing the facial motoneurons were cut into frozen sections and baked for 30 min at 37°C. The tissues were then treated with trypsin K for 25 min, rinsed with phosphate buffered saline (PBS), and the Tunel kit (35181600, Roche Group) used to stain as directed by the manufacturer: dehydrated, transparented, sealed, rinsed with PBS after staining, diaminobenzidine (DAB) stained, and hematoxylin re‐staining. To calculate the optical density, area, and proportion of all captured pictures with a positive expression, three sample sections from each animal were randomly chosen. Slices were chosen 400 times to acquire photographs with the aid of the image‐processing software Image‐Pro Plus 6.0.

### 2.9 Masson staining of buccal muscle

Sections were routinely dewaxed to water, stained with modified Masson trichrome staining solution (0311A20; Hefei Bomei Biotechnology Co., Ltd.) according to the instructions, and then they were dehydrated, transparent, sealed, and microscopically examined. Utilizing the Image‐Pro Plus 6.0 picture analysis system, the optical density and area of every collected image were measured, and the percentage of fibrous tissue expression area was computed.

### 2.10 Statistical analysis

The analysis was carried out using the SPSS 25.0 program. Mean ± SD was used to express all quantitative data. Analysis of variance was employed to compare the results of each group, and the least significant difference post hoc test was carried out. Nonparametric Wilcoxon signed‐rank test was perpormed to compare the degree of axonal ultrastructure damage. Statistics were deemed significant at *p* < 0.05.

## 3 RESULTS

### 3.1 Animal behavioral assessment

None of the animals exhibited peripheral facial palsy preoperatively, and the behavioral scores were 0. At 1 day postoperatively, rats in the MC group had a score of 5 or total facial palsy. At 7, 14, and 21 days postoperatively, different levels of facial palsy healing were seen in each MC group. At 28 days postoperatively, the behavioral scores of rats in each MC group did not reach 2 and could not be classified as facial palsy. In contrast to the NC group, all MC groups showed statistical significance at all postoperative time points (*p* < 0.05). However, there were no significant differences between the MC groups (*p* > 0.05) at any time point, as shown in Figure 1b.

### 3.2 Electrophysiologic findings

**FIGURE 2:**
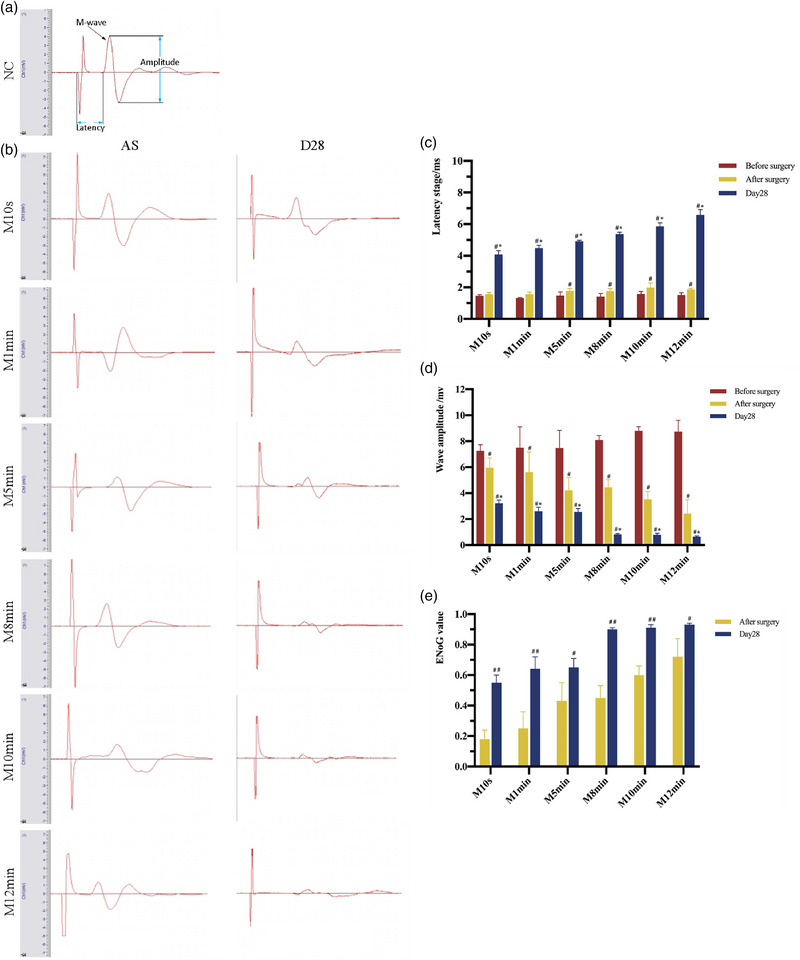
Facial nerve electrophysiology. (a and b) Electrophysiological waveforms of facial nerve in each group after surgery and 28 days postoperatively. (c) Comparison of M‐wave latency between the MC groups at different time points. *Note*: There was no significant difference of the M‐wave latency in each MC group before and after surgery. At 28 days postoperatively, the M‐wave latency increased in each MC group compared to the previous group. Compared with pre‐surgery, ^#^ *p* < 0.05; compared with post‐surgery, *p < 0.05. (d) Comparison of M‐wave amplitude between the MC groups at different time points. *Note*: The M‐wave amplitude in each MC group showed a gradual decrease in preoperative, postoperative, and 28 days postoperatively. Compared with pre‐surgery, ^#^ *p* < 0.05; compared with post‐surgery, *p < 0.05. (e) Comparison of EoG value between the MC groups at different time points. *Note*: After 28 days of surgery, the ENoG value in each MC group showed a significant increase compared to the immediate postoperative value. Only the M10min and M12min groups had ENoG values greater than 50% post‐surgery. At 28 days postoperatively, the ENoG value of each MC group was >50%, among which the ENoG values of the M10min and M12min groups were >90%. Compared with post‐surgery, ^#^ *p* < 0.05, ^##^ *p* < 0.01.

Before the surgery, there were no discernible changes between each MC group in M‐wave amplitude and latency (*p >* 0.05). All the rats were able to generate action potential from the orbicularis oris muscle after surgery and 28 days postoperatively. Except for the M10s and M1min groups, the M wave latency was longer postoperatively, and the wave amplitude was lower in the M5min, M8min, M10min, and M12min groups than that preoperatively. All of these variations were statistically significant (*p <* 0.05). At 28 days postoperatively, the M‐wave latency was substantially longer. The wave amplitude was notably reduced in each MC group when compared to that in preoperative and postoperative periods. The longer the duration of compression was, the longer the M‐wave latency and the lower the M‐wave amplitude were, with statistically significant differences (*p* < 0.01 for latency and *p* < 0.05 for wave amplitude). Only the ENoG values of M10min and M12min groups were more than 50% preoperatively. Twenty‐eight days postoperatively, the ENoG values of all the MC groups were more than 50%, among which the ENoG values of M10min and M12min groups were more than 90% (Figures 2  ).

**FIGURE 3:**
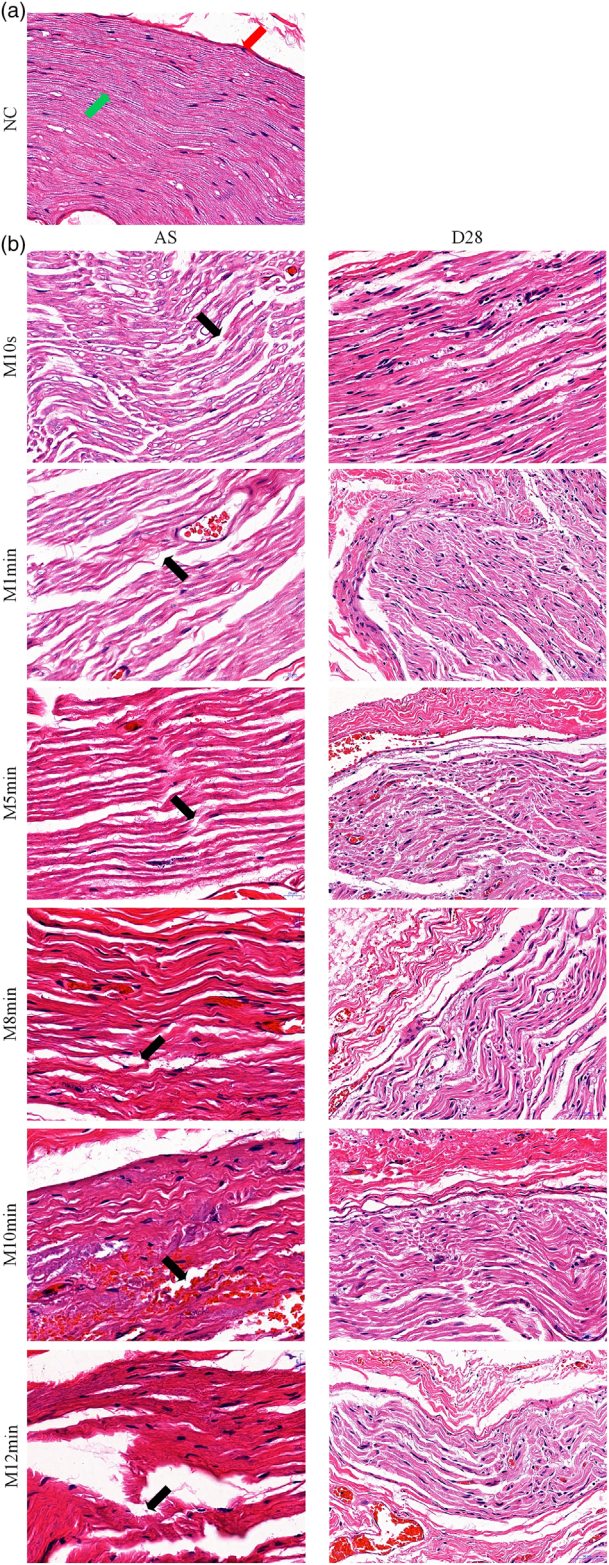
HE staining of facial nerve in each group after surgery and 28 days postoperatively. *Note*: In the normal control (NC) group, the nerve membranes were intact, and the axons were neatly arranged (a). After surgery, a series of pathological changes such as nerve fiber degeneration, necrosis, hemorrhage, nerve bundle membrane fracture, axon fracture, and myelin vacuolization appeared in each MC group (b). At 28 days postoperatively, the facial nerve damage did not recover completely. The degree of nerve damage gradually increased with the prolongation of the facial nerve compression time. The red arrow in the picture points to the outer nerve membrane, the green arrow to the axons, and the black arrows to the broken nerve fibers. Original magnification 400×; the bar indicates 50 μm.

### 3.3 Morphological analysis of facial nerve

Following the surgery, nerve bundle membrane rupture, axon dissection, and myelin vacuolization were all significantly different between the MC and NC groups under light microscopy. As the facial nerve compression period was prolonged, the severity of the injury worsened. At 28 days postoperatively, the damaged facial nerve had not fully healed in each MC group, and the lesions continued to show the aforementioned pathological alterations and gradually worsened as the compression period increased. Each MC group nearly achieved the damage degree of Sunderland IV (Figure 3).

**FIGURE 4:**
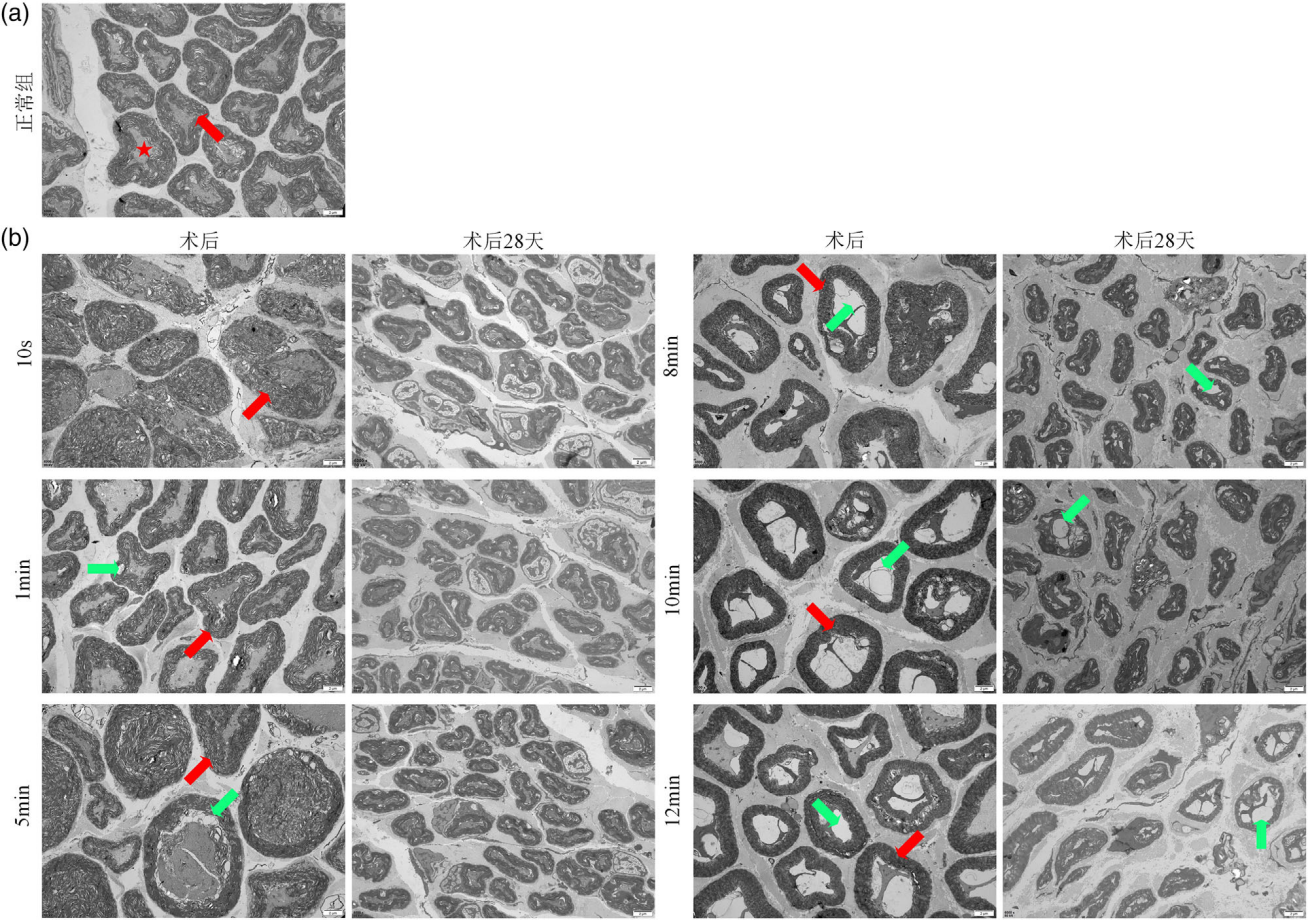
Transmission electron micrograph of facial nerves in each group 28 days postoperatively ( 6000×). *Note*: The myelin sheath and axonal structure of the facial nerve in the normal control (NC) group were normal (a). Postoperatively the varying degrees of myelin and axon injuries were visible in all MC groups (b). The M10min group and M12min group had the most severe injuries. Twenty‐eight days postoperatively, the myelin axonal structures of the M10s and M1min groups were basically normal. As the compression time increased from the M5min group, there was a greater degree of damage to myelin and axons, resulting in lesions such as myelin relaxation, axonal vacuolization, and demyelination. Myelin sheaths are indicated by red arrows, vacuolated axons by green arrows, and axons by a red star.

**FIGURE 5:**
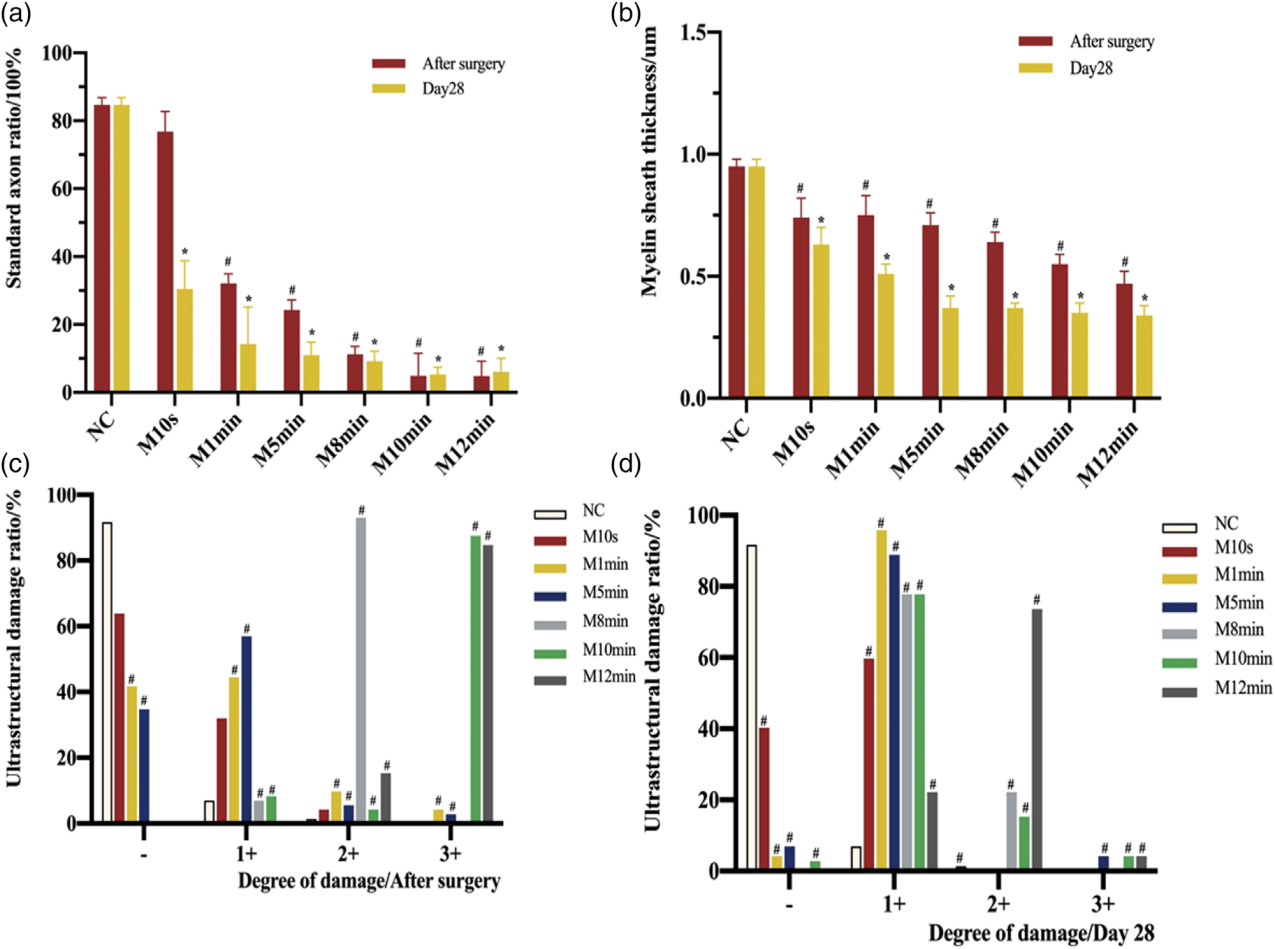
(a) Comparison of the myelin sheath thickness of facial nerve in each group after surgery and 28 days postoperatively. *Note*: In all MC groups, the myelin thickness of the facial nerve was smaller than that of the normal control (NC) group after surgery and 28 days postoperatively. Furthermore, there was a decreasing trend between each group. Compared with the NC group post‐surgery, ^#^ *p* < 0.05. Compared with the NC group 28 days postoperatively, *p < 0.05. (b) The standard axon ratio in each group after surgery and 28 days postoperatively. *Note*: In each MC group after surgery and 28 days postoperatively, the standard axon ratio was smaller than that in the NC group. There was also a decreasing trend observed between the MC groups. Compared with the NC group post‐surgery, ^#^ *p* < 0.05. Compared with the NC group 28 days postoperatively, *p < 0.05. (c and d) The ultrastructural damage ratio of facial nerve in each group under electron microscope after surgery and 28 days postoperatively. *Note*: After surgery, the extent of ultrastructural damage to the facial nerves differed among the MC groups. Additionally, the damage was more severe with longer compression times. At 28 days postoperatively, the degree of axonal and myelin lesions in the NC group was mainly concentrated in “‐,” but the lesions in each MC group were all concentrated in “1+” and “2+,” with axonal vacuolation and fissured myelin sheath. Compared with the NC group, ^#^ *p* < 0.05.

Under TEM, the myelin sheath of facial nerve trunk and morphological changes of axons were normal in the NC group, and no obvious demyelination changes were seen. In the MC group, the myelin thickness and the standard axon ratios were lower than those in the NC group both after surgery and 28 days postoperatively, and they steadily reduced as the compression period increased. At 28 days postoperatively, none of the ultrastructures in the MC groups were restored to the normal, and the myelin thickness and lesion severity varied considerably from the NC group in both of these respects (*p* < 0.05). The overall lesion degree of each MC group was concentrated in (1+, 2+): axon vacuolation, myelin delamination, and even deformation (Figures 4 and 5).

**FIGURE 6:**
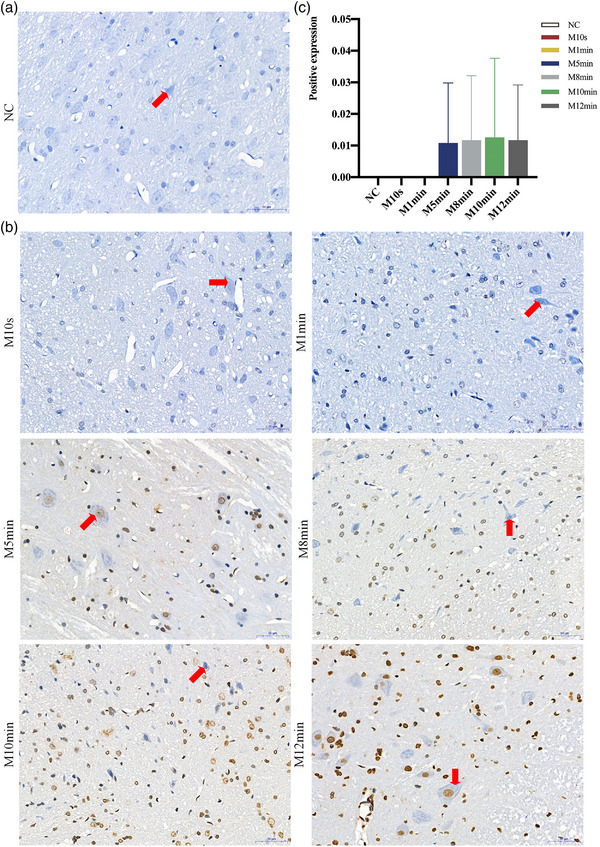
(a and b) Tunel staining of the brainstem tissue containing facial motoneurons at 28 days postoperatively (200×). *Note*: The blue or light blue staining was observed in facial motoneurons of the normal control (NC), M10s, and M1min groups, while the yellowish nuclear staining was present in individual neuronal cells of the M5min, M8min, M10min, and M12min groups. (c) Analysis of positive expression in Tunel staining of facial motoneurons at 28 days postoperatively. *Note*: The results revealed very low positive expression in the M5min, M8min, M10min, and M12min groups. However, there was no statistical significance between any of the groups (*p* > 0.05). The facial motoneurons were highlighted by the red arrows.

### 3.4 Apoptosis and Nissl body changes in facial motoneurons

In Tunel staining, the negative expression (normal nuclei) was blue or light blue, whereas the apoptotic nuclei were light yellow or brownish yellow. There was no obvious facial motoneuron apoptosis following surgery in the MC groups. Statistical significance was not observed in any of the groups (*p* > 0.05), showing that peripheral facial nerve crush injury did not significantly accelerate central facial neuron apoptosis within 28 days (Figure 6).

**FIGURE 7:**
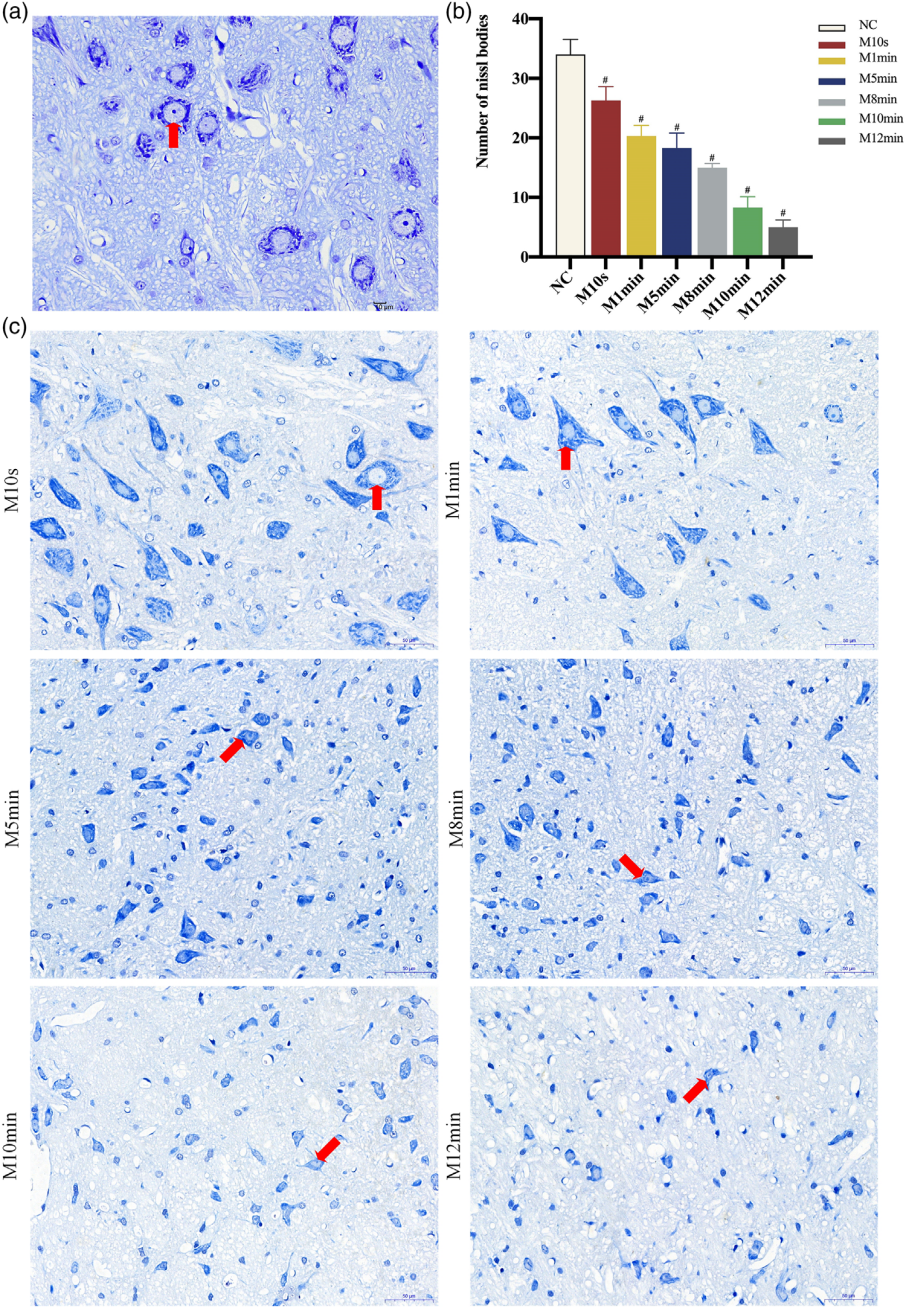
(a and c) Nissl staining of facial motoneurons in each group at 28 days postoperatively (400×). *Note*: The Nissl staining results showed that the facial motoneurons had round nuclei with nucleoli and darkly stained Nissl bodies that looked like tiger stripes in the cytoplasm. The M10s and M1min groups had nearly normal cell structures, but the M5min, M8min, M10min, and M12min groups exhibited varying degrees of damage, with fuzzy cell structures and reduced Nissl bodies in the cytoplasm. Additionally, the red arrows indicated the presence of nissl bodies in the facial motoneurons' cytoplasm. (b) Numbers of Nissl bodies in facial motoneurons in each group at 28 days postoperatively. *Note*: The study revealed that crushing the facial nerve trunk for longer periods resulted in a decrease in the number of Nissl bodies. Compared with the normal control (NC) group, ^#^ *p* < 0.05.

In Nissl staining, normal facial motoneurons had large and round nuclei with evident nucleoli and darkly colored Nissl bodies that looked like tiger stripes in the cytoplasm. The facial motoneurons in M10s and M1min groups had minor impairment 28 days postoperatively. The cytostomes of facial motoneurons in the M5min, M8min, M10min, and M12min groups were wrinkled and smaller, with hazy structures, and Nissl bodies transformed from lumpy or granular to powder‐like or even vanished from the cytoplasm of motoneurons. With the extension of the compression period, the degree of damage mentioned above rapidly grew (Figure 7).

**FIGURE 8:**
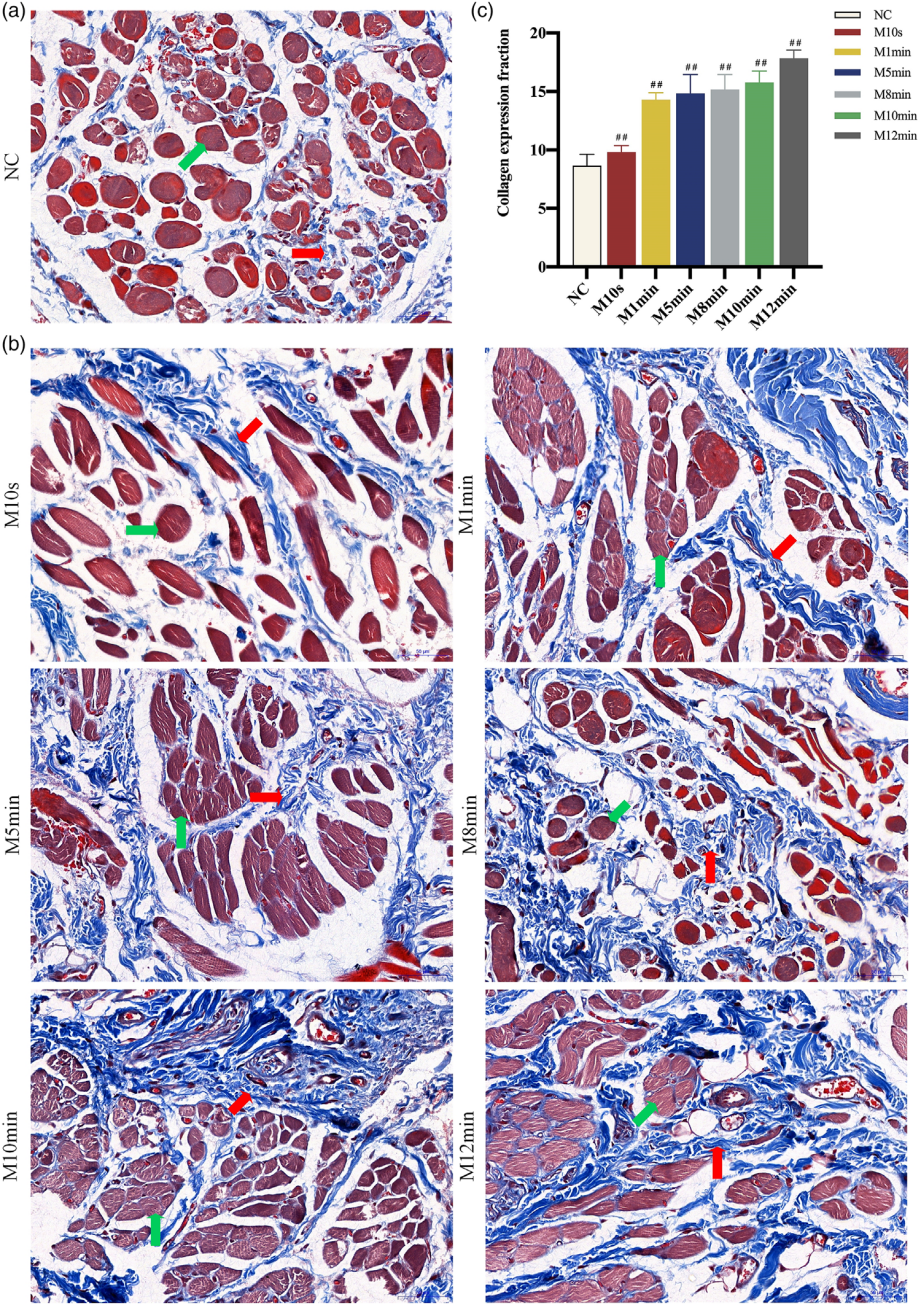
(a and b) Masson staining of buccinator in each group at 28 days postoperatively. *Note*: In the normal control (NC) group, Masson staining is manifested as red muscle staining and blue collagen fiber staining. All the MC groups experienced a decrease of buccal muscle fiber and an increase of collagen fiber at 28 days postoperatively. As the crushing time increases, the above changes become more pronounced. (c) The collagen fiber expression fraction in each group at 28 days postoperatively. *Note*: With longer pressing time, the buccal muscle showed more severe atrophy, and the area ratio of collagen fiber expression also increased gradually. Compared with the NC group, ##*p* < 0.01. In the image, the collagenous connective tissues are marked with red arrows, whereas the buccal muscle tissues are marked with green arrows.

### 3.5 Buccal muscle atrophy analysis

Masson staining of normal muscular tissue revealed that collagenous connective tissue turned blue‐purple and muscle fibers turned red. Following a muscle tissue injury, the collagenous tissue tends to become more stained as a result of scar repair or muscular fibrosis. All the MC groups experienced a decrease of buccal muscle fiber and an increase of collagen fiber 28 days postoperatively. With longer pressing time, the buccal muscle's atrophy grew more pronounced, and the area ratio of collagen fiber expression fraction gradually increased, which was notably distinct from the NC group (*p* < 0.01) (Figure 8).

## 4 DISCUSSION

The facial nerve injury model has been widely investigated for decades to assess mechanisms relating to nerve injury, survival, and regeneration as well as the assessment of neurotoxins and neurotrophic factors. It is a simple and reproducible peripheral nerve injury model (Byram et al., 2020). Nerve compression models have been extensively used to study facial nerve injury and regenerative repair pathophysiological changes. There is no agreement on the methods, locations, or nerve compression duration in the facial nerve compression models, and the self‐healing factors of the facial nerve had not been widely discussed.

The buccal branch of the facial nerve (Wang et al., 2020) or the facial nerve's main trunk (Min et al., 2022) was traditionally used to determine where the facial nerves were compressed. Compared to other branches, the buccal branch of the facial nerve was thicker and more superficial. However, unlike humans, rats had only three branches that made up the facial terminal branch: the auricular nerve, the buccal nerve, and the mandibular rim nerve (Shan et al., 2005). Additionally, some rats had variations in the buccal branch, which made it challenging to ensure consistency and reproducibility of modeling. However, the location of the facial nerve trunk was more constant and can avoid the influence of a branch innervating facial muscle movement Therefore, the main trunk of the facial nerve was more suitable for modeling. The determination of the facial nerve trunk compression time is currently controversial, ranging from 10 s (Cao et al., 2021), 30 s (Hyeok Kim et al., 2022), 1 min (Jang et al., 2014), twice for 30 s (10‐s interval) (Chen et al., 2020) to 3 min^10^, 5 min (Li et al., 2018),10 min (Li et al., 2019). Accordingly, we set up 10 s, 1 min, 5 min, 8 min, 10 min, and 12 min groups and allowed them to heal naturally. Then we used animal behavioral observation, neurophysiological testing, and tissue staining techniques to assess the damaged facial nerve regeneration.

According to Guo et al. (2016), Tang et al. (2014), and Chen and Xuan (2013), and in agreement with Simone's 10‐point technique, animal research frequently employed a facial behavioral assessment to evaluate peripheral facial palsy. Three factors were rated in this experiment to assess the function of the facial nerves: the transitory reflex, whisker movement, and nasal tip location. It was discovered by Jiang et al. (2006) and Ya et al. (2000) that although the neuropathological sections of the animals with facial nerve crush injury reached the grading criteria of facial palsy after modeling, the signs of successful modeling naturally disappeared 1 week later, which suggested the existence of the self‐healing phenomenon of the facial nerve in animals with a crush injury. Animals had a higher capacity of self‐healing than humans did, particularly mice and rats, which demonstrated the higher capacity for regeneration of the rodent nervous system (Ali et al., 2020), and the motor deficits of the nose and mouth frequently showed transient changes, with the majority of cases recovering on their own within hours or days (Joko et al., 2020). This high frequency of active movements might be the primary cause of the rapid disappearance of appearance problems because of the high frequency of nose and lip use in rats' faces. A total score of more than 3 indicated the existence of peripheral facial palsy in this trial, which employed the Simone 10‐point scale to rate the indications of facial palsy. The rats in each MC group could not achieve the animal behavioral score's diagnostic standards for facial palsy 28 days postoperatively. However, the neuropathological examination showed that the degree of facial nerve lesion was still complete facial palsy. So the animal behavioral assessment could not be used as an index to determine facial nerve healing but could be used as a macroscopic secondary criterion to evaluate the success of facial palsy model.

There was no unified standard for grading the pathology of successful facial palsy models. Most scholars used the Sunderland III degree as the standard for successful facial nerve injury modeling (Liu et al., 2017). Under the HE staining, pathological signs include bundle fracture, fuzzy nerve fiber contour, nerve fiber degeneration or necrosis, myelin sheath detachment, myelin laminae dehiscence, axonal vacuolization, and nerve endothelium fracture appeared following facial nerve injury in this experiment, which all reached at least Sunderland III degree. The damage degree increased to Sunderland III‐IV 28 days postoperatively. This was probably due to the fact that the distal nerve typically experienced Waller degeneration 48 h following facial nerve surgery. In contrast, the facial nerve trunk had not yet degenerated at this time when detected.

Rapid nerve impulse conduction relied on the myelination of nerves. The thickness of myelin sheath was closely related to myelination. As a result, the facial nerve regeneration could be evaluated based on the typical number of axons and the thickness of the myelin sheath. Under the TEM, the standard axon ratio and myelin thickness decreased with the compression time lasted. Twenty‐eight days postoperatively, the standard axon ratio and myelin thickness reduced again in the MC groups. Taking the facial nerve pathology into account, it could be said that the severity of the nerve damage increased with the pressing time lasted. As a result, the facial nerve damage was more severe 28 days postoperatively than that after surgery. Thus, the pathohistological staining performed immediately after surgery was not a reliable method for assessing the extent of facial nerve damage.

Nerve electrophysiology was one of the most sensitive and earliest indicators to determine nerve injury and repair (Bain et al., 1989), which was widely used to diagnose peripheral nerve injury. The commonly used methods were electromyography (ENoG), nerve excitability experiment, and facial nerve electrogram. In this experiment, the M‐wave latency showed an increasing tendency both immediately after surgery and 28 days postoperatively between each MC group. Additionally, the M‐wave amplitude of the MC groups showed a tendency to decline. The M‐wave latency and amplitude showed the same trend with the facial nerve trunk ultrastructural changes. The lesions were all aggravated 28 days postoperatively than that after surgery. Therefore, ENoG could quantitatively estimate the degree of nerve degeneration. However, Wallerian degeneration did not occur immediately following facial nerve injury; rather, it developed gradually from the proximal end to the distal end over roughly a week. The inflammation of the facial nerve caused early damage that could not be accurately measured through ENoG, which did not meet the necessary standards for early diagnosis. This could lead to the possibility of incorrect or missed diagnoses (Kimura, 2006). It was assumed that axonal degeneration was the leading cause of facial nerve injury within a short period. However, only the M10min and M12min groups showed ENoG values beyond 90% 28 days postoperatively, and Li et al. (2003) noted that when ENoG levels were beyond 90%, the natural healing ability of facial nerve was subpar. It is suggested that the optimal length of time for compressing the facial nerve with a 12.5 cm micromosquito clamp is a minimum of 10 min.

Following the crush surgery, the entrapment segment experienced inhibited axoplasmic transport, which prevented the target muscle from receiving muscle neurotrophic factor produced by the neuronal cytosol. This ultimately caused muscle atrophy. Masson staining was used to quantify the collagen area/bundle area value. We discovered that the buccal muscle atrophy was more apparent with the pressing time lasted. The MC groups demonstrated substantial statistical differences compared to the NC group, except for the M10s and M1min groups. The validity of the crush model has been verified again. According to the earlier studies on peripheral nerve injury, a facial nerve transection in mice causes a loss of motoneurons of 75%, while crushing the same nerve caused negligible cell apoptosis (Lee et al., 2017). Our findings were consistent with the previous investigation, which found no evidence of significant central facial motoneuron apoptosis following facial nerve trunk crush injury for varying lengths of time. Even cytosolic crinkling and deformation were visible in Nissl staining, demonstrating pathomorphological alterations in facial motoneurons. Cytoplasmic Nissl bodies gradually changed from blocky to granular, then to powdery or even disappeared. With the lengthening of the pressing time, the severity of the damage mentioned above gradually grew.

In summary, the study supported the possibility of using animal behavioral evaluation as an additional criterion to assess the early‐stage effectiveness of the facial nerve crush damage model. The ENoG could qualitatively identify facial nerve damage and assessed the extent of nerve fiber degeneration. Still, it could not identify the ultrastructural damage to the facial nerve. The ideal duration to compress the facial nerve by utilizing a 12.5 cm micromosquito clamp as a facial palsy model was at least 10 min, which could ensure that the facial nerve injury met the criteria for complete facial palsy and the ENoG value was beyond 90% beyond. This greatly reduced the likelihood of complete self‐healing of the facial nerve, and created a significant difference from the NC group, ensuring the impartiality of subsequent experimental results.

This study also had several limitations worthing further efforts. Referring to our study, to exclude the influence of facial nerve self‐healing factors on the next experiment, we needed to press the facial nerve trunk for at least 10 min. Compared with the previous literature, the time was relatively longer. We assumed that under the premise of achieving the same degree of nerve damage, whether the pressing tool or the pressing force could be changed to shorten the pressing time. Moreover, what factors affected the self‐healing of facial nerves and its molecular mechanisms needed to be further studied.

## AUTHOR CONTRIBUTIONS

Jing Fei designed the study and drafted the manuscript. Xirui Guan and Hongdi Zheng performed animal surgery, histology, and morphometry experiments. Lin Gao and Ping Ni collected the data. Lin Gao and Kunling Duan analyzed and interpreted the data. Na Liao and Leiji Li conceived and designed the experiment and edited the review. All authors were involved in drafting and revising the manuscript. All authors approved the final version that was accepted for publication.

## CONFLICT OF INTEREST STATEMENT

The authors declare no conflict of interest.

### PEER REVIEW

The peer review history for this article is available at https://publons.com/publon/10.1002/brb3.3156.

## Data Availability

The data that support the findings of this study are available from the corresponding author upon reasonable request.
